# A Case Series on the Efficacy of the Pharmacological Treatment of Lipedema: The Italian Experience with Exenatide

**DOI:** 10.3390/clinpract15070128

**Published:** 2025-07-07

**Authors:** Laura Patton, Valeria Reverdito, Alessandra Bellucci, Micaela Bortolon, Annalisa Macrelli, Lorenzo Ricolfi

**Affiliations:** 1 Endocrinology and Lymphology Clinic, 38096 Vallelaghi, TN, Italy; lorenzoricolfi@hotmail.it; 2 Physiotherapy Clinic, 10138 Torino, TO, Italy; valeria.reverdito@gmail.com; 3 Nutrition Clinic, 38066 Riva del Garda, TN, Italy; alessandrabellucci14@gmail.com; 4 Rehabilitation Unit and Lymphology Clinic, Institute San Gregorio, 31049 Valdobbiadene, TV, Italy; micaelabortolon@gmail.com; 5 Physiotherapy Clinic, 47039 Savignano sul Rubicone, FC, Italy; macrelli.annalisa@gmail.com

**Keywords:** exenatide, lipedema, glucagon-like peptide-1 agonist receptor, drug therapy, insulin resistance, inflammation, adipose tissue, very-low-calorie ketogenic diet, pain, ultrasound

## Abstract

**Background:** Lipedema is a chronic disease of subcutaneous adipose tissue that predominantly affects women and is frequently associated with endocrinopathies such as insulin resistance and obesity. Its pathogenesis is still unclear, and treatment, which requires a multi-disciplinary approach, is prolonged over time and is not always effective. There is currently no drug treatment available for this disease. **Methods:** Five different cases of women with lipedema and insulin resistance, treated with Glucagon-Like Peptide-1 Receptor Agonists (GLP-1 RAs) and once-weekly exenatide, in association or not with lifestyle changes (diet or physical activity) for 3 to 6 months are described. Changes in anthropometric parameters, symptoms, clinical findings and the thickness of superficial adipose tissue measured by ultrasound were evaluated. **Results:** Treatment with exenatide, whether combined with a change in diet or physical activity, resulted in a reduction in the characteristic symptoms of lipedema, in pain evoked by pinching the adipose tissue fold and in the thickness of subcutaneous adipose tissue at the levels of the lower limbs, abdomen and upper limbs. In four out of five cases, a reduction in body weight was observed, particularly during the first three months of treatment and in cases with greater metabolic impairment. Clinical, instrumental and subjective improvements were also observed in cases where there was no reduction in body weight and in patients who had previously undergone lower limb liposuction. **Conclusions:** The improvement in symptoms and clinical signs of lipedema, in addition to the reduction in adipose tissue in patients with lipedema and insulin resistance with exenatide, suggests a novel pharmacological approach to the disease, which can be combined with other conservative and surgical treatments to promote weight reduction. These results also highlight the association of this disease with metabolic alterations and the fundamental role of an accurate diagnosis followed by the treatment of comorbidities and excess weight in these patients.

## 1. Introduction

Lipedema is a chronic disease of the subcutaneous adipose tissue that is still poorly understood by health professionals. For this reason, it may be difficult to diagnose and, consequently, difficult to treat due to the lack of scientific data and training of the many professionals who should be involved in the care of patients. The great lack of scientific support is not justified by the rarity of the disease, as is unfortunately often the case with many lesser-known diseases, given that prevalence estimates range from 6.5% in children in the United States to 6–8% in women in Germany and 15–19% in clinics specializing in vascular diseases [1]. While experiences with conservative treatments, such as physiotherapy, diet and/or surgery, are beginning to be shared and obtain a scientific basis, the same cannot yet be said for a possible pharmacological approach. In fact, there is currently no data available, not even on pain control, which is one of the main symptoms of the disease.

In a recent publication of ours, we noted the presence of numerous comorbidities in women with lipedema, many of which involve the endocrine system [2]. Among the most common comorbidities we found were obesity, alterations in glucose metabolism, dyslipidemia, thyroid disorders and polycystic ovary syndrome. If it is true that there is no defined therapy for lipedema, it is also true that for these comorbidities, there is a long history of scientific experience and proven efficacy. Our data suggests that at least one third of women with lipedema are affected by insulin resistance; this means that at least one third of lipedema patients could benefit from targeted pharmacological treatment. This percentage becomes over 50% in women with both lipedema and obesity, while it rises to 75% if BMI is greater than 35 kg/m^2^ [2].

Insulin resistance, due to its implications on metabolism, adipocytes and inflammation, could reasonably be implicated in the pathogenesis and progression of adipose tissue diseases such as lipedema. Therefore, based on this scientific evidence, a treatment aimed at improving this condition could potentially have many favorable effects [2,3,4,5,6,7,8]. The results obtained with some nutritional regimens, such as the ketogenic diet or a low-carb and anti-inflammatory diet, which have an impact on glucose metabolism, have already proven to be effective in treating lipedema [9,10,11,12,13,14,15,16,17,18].

Metformin remains one of the cornerstone treatments for insulin resistance, even in other endocrinopathies such as polycystic ovary syndrome, where its pleiotropic effects and its role in the treatment of this syndrome are well known [19,20,21]. Treatment with metformin is also a valid option for patients with lipedema and insulin resistance and is, in fact, part of our daily clinical practice regarding this metabolic condition.

But Glucagon-Like Peptide-1 Receptor Agonists (GLP-1 RAs) may represent an even more interesting pharmacological option for women with lipedema and insulin resistance. In fact, to treat insulin resistance in these patients, for the past year, we have also been using one of the first drugs of the GLP-1 RA family that was introduced for the treatment of diabetes about 20 years ago in Italy; at the time, it was considered an innovative therapy with promising properties because improvements in glucose control were often accompanied by a reduction in bodyweight [22,23].

Type 2 diabetes mellitus is currently the only indication authorized for the prescription of exenatide, but there is a lot of data in the literature demonstrating the drug’s ability to improve hepatic, adipose and whole-body insulin sensitivity while having positive effects on body weight reduction, both in diabetic and non-diabetic patients [24,25]. In preclinical and clinical studies, exenatide has been demonstrated to improve glycemic control through the enhancement of glucose-dependent insulin secretion, the suppression of inappropriately elevated postprandial glucagon secretion, the slowing of gastric emptying and the reduction in food intake [26,27,28,29].

Although pharmacological intervention on insulin resistance and weight loss in these patients may be extremely important, there are other demonstrated actions of this pharmacological class that are well beyond the treatment of the metabolic disorder. More and more studies have emphasized the therapeutic potential of GLP-1RAs in a wide variety of diseases, such as neurodegenerative disorder and inflammatory bowel disease, shedding light on their anti-inflammatory effects [30]. Exenatide, specifically, was found to reduce oxidative stress and the expression of numerous pro-inflammatory cytokines, such as, for example, monocyte chemoattractant protein 1 (MCP-1), Tumor Necrosis Factor alpha (TNF-alpha), and Interleukin-1 beta (IL-1 beta), in obese patients [31,32,33]. The anti-inflammatory effect at the cellular and molecular levels was independent of weight loss [33]. Other studies have shown the effect of exenatide on reducing inflammation and hypoxia in adipose tissue by improving angiogenesis and microcirculation [34]. These effects, which are fundamental for the treatment of simple obesity, take on an even more potentially interesting importance when considered for the treatment of a disease such as lipedema, whose main characteristic seems to be the presence of inflamed, hypo-oxygenated and dysfunctional adipose tissue with fibrosis, extracellular matrix remodeling and lymphatic and vasculature dysfunction [1,35].

The aim of this study, with these assumptions, is to present the first promising evidence of the efficacy of exenatide Long-Acting Release (exenatide LAR) in treating the signs and symptoms of five women affected by lipedema and insulin resistance.

## 2. Materials and Methods

Five cases of patients with lipedema treated for 3 to 6 months with exenatide LAR 2 mg per week in association or not with lifestyle changes (diet or physical activity) are described. During the study period, no other conservative treatments were performed, such as the use of bandages, lymphatic drainage or elastic compression garments (except for case 5, as described below).

The inclusion criteria were the presence of lipedema, female sex, age between 18 and 50 years and the presence of insulin resistance.

The exclusion criteria were the presence of diabetes mellitus, serious chronic diseases, the intake of drugs with any effect on body weight, glucose metabolism, pain and edema. Patients with a recent reduction in body weight (greater than 10% of the weight in the previous 6 months) or diet modification or patients previously treated for lipedema (including methods such as subcutaneous adipose tissue massage, lymphatic drainage, bandaging or elastic compression stocking) were also excluded. Case report 5 is an exception as the patient had undergone previous surgical therapy and was using elastic compression only for this reason: this treatment was not modified during the study and in the 6 months before the study. Informed consent was obtained from all subjects for the use of off-label exenatide therapy and for the publication of the results.

The diagnosis, staging and phenotyping of lipedema was based on clinical findings of characteristic symptoms and signs of the disease, as described in our previous work [2,36,37].

The presence of insulin resistance was assessed both in fasting and after the ingestion of 75 g of glucose. All patients performed the Oral Glucose Tolerance Test (OGTT), with blood glucose and insulin levels being measured before and 30, 60, 90, 120 and 180 min after glucose ingestion. The fasting insulin resistance status was determined by calculating the HOMA-IR index (HOMA-IR = (glucose mg/dL × insulin mIU/L)/22.5) [38]; a value greater than or equal to 2.29 was considered diagnostic for the presence of fasting insulin resistance [39].

As a second method for the diagnosis of insulin resistance, the surrogate index called the Matsuda Index was used [40]; a value of less than 3.5 was considered diagnostic for insulin resistance [41].

Diagnostic criteria for the diagnosis of prediabetes were as follows: fasting glucose of 100–125 mg/dL, 120 min OGTT glucose of 140–190 mg/dL or HbA1c 5.7–6.4% [42].

The presence of diabetes was excluded according to Italian and international guidelines [43,44].

No specific questionnaires were used to evaluate diet and physical activity, which were assessed by nutritionists and doctors during the visits. The recommended diet, in case reports from a retrospective observational study, could be different, as described in individual cases. Generally, during the pharmacological treatment phase, moderate aerobic physical activity is recommended, such as walking or gym exercises for 30–60 min a day at least three times a week.

No assessment of body composition was performed, although patients were evaluated using anthropometric measurements (such as body weight, BMI and waist and hip circumference), a specific questionnaire for lipedema symptoms, a Progressive Pain Check for lipoalgia and an ultrasound assessment of the adipose tissue.

### 2.1. Evaluation of Lipedema Symptoms

For the evaluation of symptoms we used a specific questionnaire, as described in our previous work [36]. The questionnaire was used to evaluate the severity of symptoms before beginning therapy and after therapy and consists of 17 questions that investigate the presence and extent of the main symptoms of the disease. To quantify the extent of symptoms, a 6-point Likert-type scale was used (0 = none, 1 = very mild, 2 = mild, 3 = moderate, 4 = strong and 5 = very strong) [45].

Symptoms can be assessed individually, and a final score can be calculated from the sum of individual scores, ranging from 0 to 85 [36].

### 2.2. Evaluation of Lipoalgia

The presence and extent of pain from the subcutaneous fat fold, or provoked lipoalgia, were assessed using a method called Progressive Pain Check (PPC), described in our previous work [36]. The clinical examination involves the assessment of pain evoked by the subcutaneous fat fold at 8 points on the lower body (7 points at the level of the lower limb and 1 point at the level of the lower abdomen) and at 3 points on the upper body (dorsal region, arm and forearm). Pain is quantified with the VRS, which is represented by a 5-point Likert-type scale with 5 verbal descriptors: no pain, mild pain, moderate pain, severe pain and very severe pain [36].

The quantification of pain severity was achieved through the assignment of numerical values to the VRS items, ranging from 0 to 4. These values are defined as follows: 0 = no pain, 1 = mild pain, 2 = moderate pain, 3 = severe pain and 4 = very severe pain. Subsequently, three scores were calculated: (the Lower Body Pain Score (LBPS), the Upper Body Pain Score (UBPS) and the Total body pain score or Ricolfi–Patton Score (RPS), as described in our previous work [36].

### 2.3. Ultrasound Measurement

Ultrasound measurement of the adipose tissue was conducted using a high-frequency linear probe with a frequency range of 8–14 MHz (SonoScape X3, SonoScape Medical Corp., Shenzhen, China). The probe was maintained in a perpendicular position relative to the skin and no pressure was applied to the underlying tissue.

At the level of the lower limbs, as described in our previous studies [2,36], the measurement of the subcutaneous adipose tissue was taken from the surface of the skin to the muscle fascia. The measurements were performed at the levels of the lower limb, lower abdomen, arm and forearm. At the lower-limb level, the measurements were taken at these points:

- The medial and lateral lower third of the leg (about 5 cm above the medial and the lateral malleolus);
- The medial and lateral upper third of the leg (about 5 cm below the prominence of the tibial tuberosity and an equivalent point on the lateral upper third);
- The medial and lateral lower third of the thigh (about 5 cm above the upper edge of the patella);
- The medial upper third of the tight (about 5 cm below the inguinal crease).

The following points have also been added to the lower limb:

- The midpoint of the thigh on the anterior side, corresponding to the point where the skin fold is generally detected [46,47].

Measurements were also taken at the level of the abdomen and upper limb.
At the level of the abdomen (anterior surface and with the patient supine), measurements were taken using the navel as a reference point:

- At the point 5 cm lateral and inferior to the umbilicus (lower abdomen)
- At the point 5 cm lateral and superior to the umbilicus (upper abdomen)

Measurements at the upper-limb level were taken with the patient lying down and the arm raised above the head: in this position, we took two measurements of adipose tissue thickness, namely at the midpoint of the upper arm and at the midpoint of the forearm (midpoint upper arm and midpoint forearm).

## 3. Case Series

The clinical and biochemical features of the five cases described in this manuscript are summarized in Table 1. All patients are women affected by lipedema of the lower and upper limbs in different clinical states and are affected by insulin resistance and obesity. In all cases, lipedema involved the entire lower limb from the pelvis to the ankle. In all cases, the upper limb was also involved, but in some cases, lipedema involved the arm and forearm, while in others, only the arm was involved.

Fasting insulin resistance, assessed with the Homeostasis Model Assessment of Insulin Resistance (HOMA-IR), was present in all five cases. Insulin resistance to the 75 g Oral Glucose Tolerance Test (OGTT), assessed with the surrogate Matsuda Index, was present in four cases. Case 1 is the case that had only fasting insulin resistance and not resistance to OGTT. Pre-diabetes was present in four out of five cases (Table 1). None of the patients had diabetes mellitus.

### 3.1. Case Report 1

#### 3.1.1. Description

The first case is a 42-year-old woman diagnosed with lipedema of the lower limbs (stage 2, type 3) and upper limbs (stage 1, type 4A) that began with pubertal development and gradually worsened over the years with weight gain (Table 1). No significant clinical worsening was reported after hormonal treatments for infertility or with pregnancies. The patient was of normal weight at the time of menarche but reported developing progressive weight gain over the years, especially in the last 3–4 years, up to a maximum weight of 89 kg, the weight at the time of our evaluation.

The patient had menarche at the age of 13 years and is affected by polycystic ovary syndrome characterized by the presence of severe oligo-menorrhea (4–6 cycles/year from menarche to pregnancy), micro polycystic ovary and the absence of hyperandrogenism. Regarding infertility, the patient had undergone medically assisted reproduction and had two full-term pregnancies without medical complications, with the first being at the age of 35 and the second at the age of 38.

Previously performed blood tests showed the presence of fasting hyperglycemia and normal thyroid function. At the time of our evaluation, the patient was not undergoing any pharmacological therapy.

The results of the blood tests we performed were as follows: fasting hyperglycemia and fasting insulin resistance were confirmed, with normal blood glucose and insulin response to glucose load; androgens, prolactin and cortisol levels were normal. Thyroid function was normal with normal levels of thyroid peroxidase and thyroglobulin antibodies while vitamin D deficiency was found.

#### 3.1.2. Treatment

Given the finding of insulin resistance, the patient was treated with exenatide LAR 2 mg/week for 3 months in combination with moderate physical activity (2 h/week). During these 3 months, the patient did not change her dietary habits.

#### 3.1.3. Results

The drug therapy was well tolerated, and the patient did not experience any side effects. The patient reported a decrease in appetite during treatment.

After 3 months, we did not observe any changes in body weight, while there was a reduction in waist and hip circumference (Table 2).

Symptoms in the lower limbs were attenuated; she reported a reduction in pain evoked by touch and ease of bruising and the total score of the symptom questionnaire was reduced.

Clinical evaluations showed an improvement in tissue consistency and a reduction in pain scores that was more pronounced at the levels of the abdomen and lower limbs, except for the arm, where it increased.

**Figure 1:**
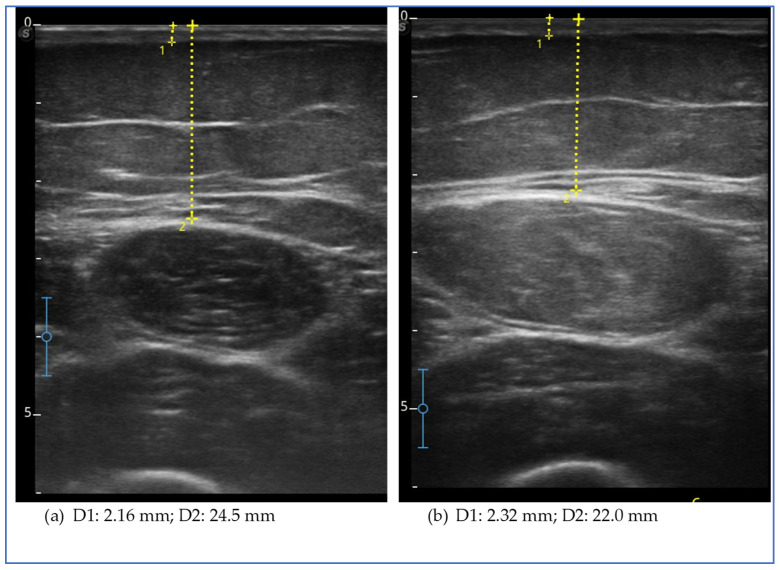
Ultrasound measurements for case report 1.

Ultrasound evaluation showed a reduction in subcutaneous fat tissue thickness in the lower extremities, in the abdomen and in the upper extremities, except for the arm, where it increased (Figure 1).

The figure shows the ultrasound measurements at the beginning of the study (a) and at the end of the study (b) of case report 1. The images refer to the anterior middle third of the thigh. The yellow dotted line represents the measurement of the thickness of the skin, which includes the dermal and epidermal layer (D1), and of the subcutaneous adipose tissue (D2), which includes all the tissue from the skin to the muscular fascia of the rectus femoris.

### 3.2. Case Report 2

#### 3.2.1. Description

The second case is a 41-year-old woman who was diagnosed with lipedema of the lower limbs (stage 2, type 3) and upper limbs (stage 2, type 4A), which began with puberty and progressively worsened over the years following weight gain (Table 1). Lipedema did not worsen following estrogen–progestin therapy, which she took for about 6 months in adolescence. The patient was of normal weight until the age of 20 when, after a reduction in physical activity and poor attention to diet, she began to gain weight progressively, especially in the past 3–4 years, when her weight reached it maximum of 91 kg at 39 years of age.

Additional information on the clinical history is as follows: the patient had menarche at 10 years of age and had a regular menstrual cycle; in adolescence, she reported a diagnosis of polycystic ovary syndrome, no longer found in adulthood, with the absence of clinical or biochemical hyperandrogenism; and she has not had any pregnancies. She had been suffering from hypothyroidism for about 18 years and was undergoing levothyroxine replacement treatment. She suffered from gastroesophageal reflux and had undergone cholecystectomy for gallstones many years earlier.

Previous blood tests showed the presence of fasting hyperglycemia.

The results of the blood tests we performed were as follows: the presence of fasting hyperglycemia was confirmed; fasting insulin resistance and normal glycemic and insulin responses to glucose load were found; and androgen, prolactin and cortisol levels were normal. Thyroid function was normal with normal levels of thyroid peroxidase and thyroglobulin antibodies. Vitamin D deficiency was noted.

#### 3.2.2. Treatment

The patient was treated with exenatide LAR 2 mg/week for 3 months in combination with moderate physical activity (3 h/week). During this period, she reduced the consumption of sweets and refined sugar.

#### 3.2.3. Results

The patient was re-evaluated after 3 months of exenatide therapy. She had no side effects.

At the end of 3 months, she reported a reduction in lower limb discomfort and an improvement in tissue consistency. The variation in the score of the questionnaire confirmed the improvement in symptoms, with a reduction in the total score and in each individual item (Table 3).

Clinical evaluations showed a weight loss of 5 kg and a reduction in pelvic circumference, while waist circumference was unchanged.

In the evaluation of evoked pain with skinfold, a reduction from the knee to the thigh and at the level of the upper limbs was noted. All three scores, namely those for lower body, upper body and RPS, improved.

**Figure 2:**
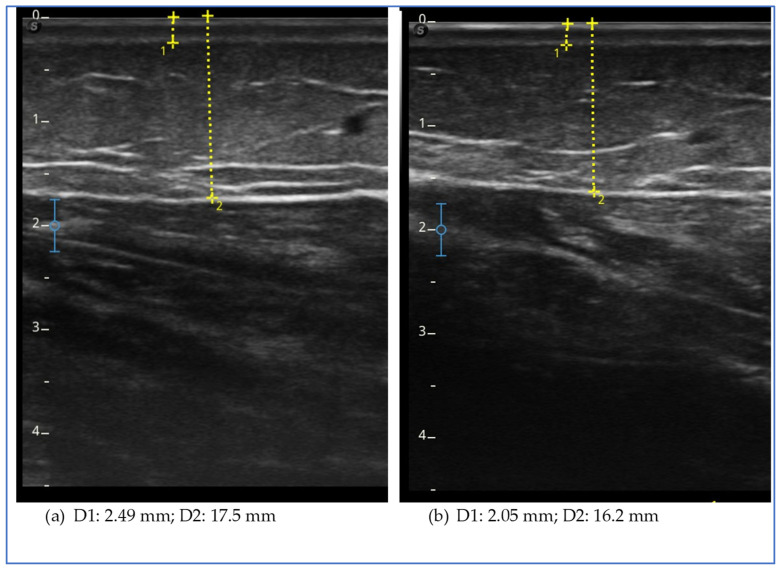
Ultrasound measurements for case report 2.

Ultrasound evaluation showed a reduction in the thickness of the subcutaneous adipose tissue in all assessed levels of the lower and upper limb, except the lateral lower third of the thigh (Figure 2). We observed a reduction in thickness in the lower abdomen but an increase in the upper abdomen.

The figure shows the ultrasound measurements at the beginning of the study (a) and at the end of the study (b) for case report 2. The images refer to the lower medial third of the leg. The yellow dotted line represents the measurement of the thickness of the skin, which includes the dermal and epidermal layers (D1), and of the subcutaneous adipose tissue (D2), which includes all the tissue from the skin to the muscular fascia.

### 3.3. Case Report 3

#### 3.3.1. Description

The third case is a 37-year-old woman diagnosed with lipedema of the lower limbs (stage 3, type 3) and upper limbs (stage 2, type 4A) that began in childhood and did not worsen with puberty but worsened over the years with weight gain and the use of estrogen–progestin therapy (Table 1). The patient reported an onset of weight gain in childhood, with overweight at the time of menarche and a long history of weight fluctuation throughout her life, reaching a maximum weight of 97 kg at the time of our evaluation.

Additional information on the clinical history is as follows: she had menarche at 11 years of age and a regular menstrual cycle; at the time of the evaluation, she was using a levonorgestrel-releasing intrauterine device (LNg IUD) for contraception; and she had a full-term pregnancy at 25 years of age, regular. She reported previous history of recurrent shoulder dislocation (Beighton Score, evaluated for assessing joint mobility, was not indicative of hypermobility).

Previous blood tests showed the presence of insulin resistance.

The results of the blood tests we performed confirmed the presence of insulin resistance in the fasting state and after the 75 g glucose load with a normal glycemic curve. Thyroid function was normal with negative antibody research. Vitamin D deficiency was found.

#### 3.3.2. Treatment

The patient was treated with exenatide LAR 2 mg/week for 6 months in combination with moderate physical activity (3 h/week) and an anti-inflammatory, gluten-free, lactose-free, low-carb and hypocaloric diet.

#### 3.3.3. Results

The patient was re-evaluated by telephone contact after 3 months and by clinical evaluation after 6 months. She had no side effects, except for mild nausea during the first month of treatment. After the first 3 months of therapy, she reported a weight reduction of 4.3 kg. After 6 months of therapy the patient showed a weight loss of 6 kg and a reduction in waist and hip circumference (Table 4). She reported an improvement in all lower limb complaints, particularly pain, swelling and ease of bruising. Symptom questionnaire scores confirmed a reduction in symptoms.

Evoked lipoalgia with skinfold assessment showed a reduction in pain at all sites except for the lower medial third of the leg, with a reduction in all three scores, namely the lower and upper body and total.

**Figure 3:**
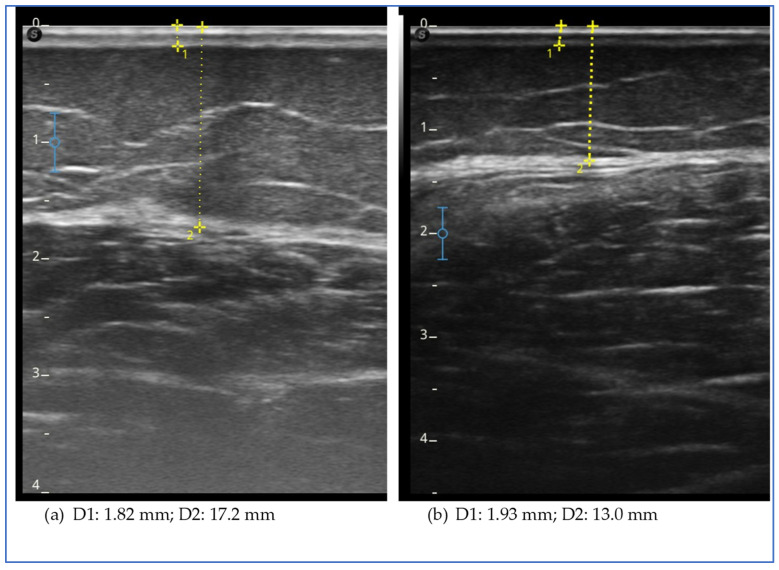
Ultrasound measurements for case report 3.

The ultrasound evaluation showed a reduction in the thickness of the subcutaneous adipose tissue in all sites assessed of the lower and upper limbs and lower abdomen, with the exception of the lower lateral third of the leg (Figure 3).

The figure shows the ultrasound measurements at the beginning of the study (a) and at the end of the study (b) for case report 3. The images refer to the upper lateral third of the leg. The yellow dotted line represents the measurement of the thickness of the skin, which includes the dermal and epidermal layers (D1), and of the subcutaneous adipose tissue (D2), which includes all the tissue from the skin to the muscular fascia.

### 3.4. Case Report 4

#### 3.4.1. Description

The fourth case is a 43-year-old woman diagnosed with lipedema of the lower limbs (stage 2, type 3) and upper limbs (stage 3, type 4A), which occurred at the age of 30 after the first cycle of ovulation induction treatment for infertility. Since then, there was progressive weight gain over the years to a maximum weight of 87.3 kg at the time of our assessment (Table 1). Over the years, she reported that she had tried several diets, including ketogenic diets, with little to no effect on body weight or on the reduction in lipedema symptoms.

Additional information on the clinical history is as follows: the patient had menarche at 10 years of age and a regular menstrual cycle; she had two full-term pregnancies, after ovulation induction for infertility, without complications at 31 and 37 years of age; and she had a diagnosis of celiac disease, and thrombophilia and dyslipidemia were observed. She reported complications from Severe Acute Respiratory Syndrome Coronavirus 2 infection (long COVID syndrome) with cardiovascular manifestation (arterial hypertension and tachyarrhythmias) and diffuse clonus, which resolved in a few months. The patient suffered from anxiety-depressive syndrome with pharmacological support. At the time of our evaluation the patient was taking the following medications: fluoxetine, rosuvastatin, ezetimibe and mirtazapine.

The results of the blood tests were as follows: we found the presence of insulin resistance on fasting and with OGTT, and we found fasting hyperglycemia and impaired glucose tolerance on OGTT.

The levels of gonadotropin, estrogen and progesterone were normal, as well as the levels of androgen, cortisol, ACTH and thyroid function.

We found the presence of anti-thyroid peroxidase antibodies (Hashimoto’s thyroiditis) and vitamin D deficiency.

#### 3.4.2. Treatment

The patient was treated with exenatide LAR 2 mg/week for 6 months. In the first 3 months of pharmacological treatment with exenatide, the patient followed a very-low-calorie ketogenic diet (VLCKD), followed by 3 months of gradual reintroduction of complex carbohydrates (30 g/day the first month, and then 40 g/day) and increased calories (from VLCD to hypocaloric diet). Because of her coeliac condition, gluten was always excluded from the diet. She did not change her physical activity levels.

#### 3.4.3. Results

The patient was re-evaluated after 3 and 6 months of treatment. The drug therapy was well tolerated. During the ketogenic diet, she reported having panic attacks (in 2–3 episodes), a pre-existing condition but temporarily aggravated, for which she was re-evaluated by her psychiatrist. No changes in drug therapy were necessary. Subsequently, she did not have similar episodes, and her mood improved.

Body weight decreased by about 10 kg in the first 3 months and then gradually increased again with the change in diet in the last 3 months (+7.3 kg) (Table 5).

The symptoms reported in both the lower and upper limbs improved from the first months of treatment and progressively during treatment, even with the increase in body weight. At the end of the 6-month therapy period, the patient reported complete remission of spontaneous pain, tenderness and of the tendency to bruise both in the lower and upper limbs.

**Figure 4:**
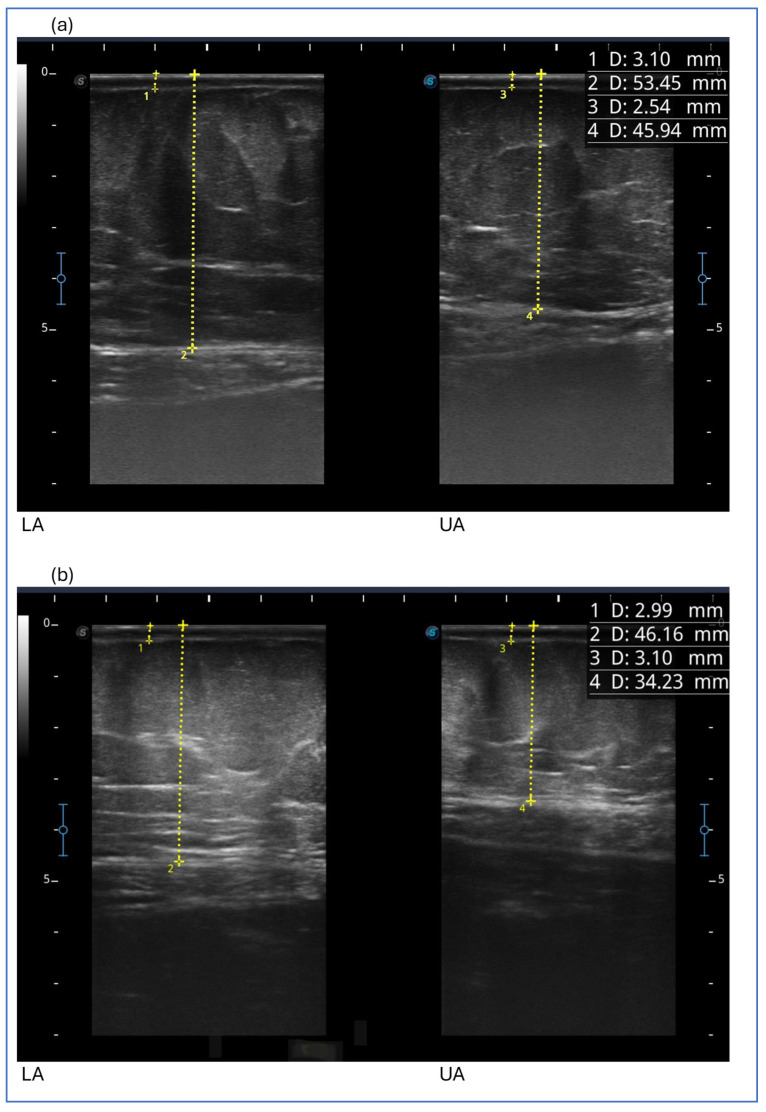
Ultrasound measurements for case report at the beginning of the study in the image above (**a**) and at the end of the study in the image below (**b**) for case report 4.

After the first 3 months, we detected a reduction in the thickness of subcutaneous adipose tissue at all levels examined in the lower limb, abdomen and upper limb. Then, the thickness of subcutaneous adipose tissue generally increased, with the exception of the upper anterior third of the thigh and upper abdomen, which showed a further reduction. It remained unchanged at the two points of the lower limb (medial upper third of the leg and anterior middle third of the thigh) (Figure 4).

After 6 months of therapy, despite weight loss and regain, we observed a reduction in the thickness of the adipose tissue at all levels of the lower and upper limb and the abdomen, except for the medial lower third of the thigh and forearm.

The lipoalgia score after the first 3 months was reduced at all sites except the medial upper third of the thigh and was not elicited at any site after the additional 3 months of therapy despite the re-increase in body weight and subcutaneous tissue thickness. Even the pain that had not reduced at the medial upper third of the thigh disappeared at the end of the 6 months of therapy.

The figure shows ultrasound measurements at the beginning of the study in the image above (a) and at the end of the study in the image below (b) for case report 4. The images refer to the lower abdomen (LA) and upper abdomen (UA). The yellow dotted line represents the measurement of the thickness of the skin, which includes the dermal and epidermal layers (1 D and 3 D), and of the subcutaneous adipose tissue (2 D and 4 D), which includes all the tissue from the skin to the muscular fascia.

### 3.5. Case Report 5

#### 3.5.1. Description

The fifth case concerns a 45-year-old woman suffering from lipedema of the lower and upper limbs (Table 1). This patient had already undergone three liposuction operations on the lower limbs at the time of our evaluation (5, 4 and 3 years before): the first operation was performed at the age of 40 on the ankles and knees (power-assisted liposuction and water-jet-assisted liposuction), the second at the age of 41 in the anterior region from the knee to the thigh (power-assisted liposuction, vibration amplification of sound energy at resonance-assisted liposuction and Renuvion skin tightening by J-Plasma) and the third at the age of 42 in the posterior region from the knee to the buttocks (power-assisted liposuction, vibration amplification of sound energy at resonance-assisted liposuction and Renuvion skin tightening by J-Plasma). The patient had very good results with the surgery. She had always worn elastic compression garments on the lower limbs and underwent maintenance treatments regularly. The results of the surgery were maintained, until for personal reasons with lifestyle changes, she presented an increase in body weight by about 10 kg, which worsened the clinical situation both in the treated areas with recurrence of pain and in areas not treated surgically, like the abdomen, thorax and upper limbs.

At the time of our evaluation, the patient presented with lipedema of the lower limbs (stage 1, type 3), upper limbs (stage 2, type 4C) and abdomen and had painful and increased consistency pathological tissue at the level of the anterior thorax.

Lipedema in this patient had its onset at the age of 18 years, in association with the use of estrogen–progestin contraceptives (continued for 7 years) and progressive weight gain (approximately 10 kg in 3 years), reaching a maximum weight of 83 kg at the age of 29 years.

Additional information on the clinical history is as follows: the patient had menarche at 10 years of age, an always regular menstrual cycle and two full-term pregnancies at the ages of 25 and 27 without complications. Uterine myomas were reported. She also reported a history of depressed mood.

At the time of our evaluation, the patient was not undergoing any pharmacological therapy.

The results of the blood tests we performed showed fasting insulin resistance with normal fasting blood glucose. At the OGTT, insulin resistance was present with marked hyperinsulinemia in response to the glucose load.

The levels of gonadotropins, estrogens and progesterone were normal, as well as the levels of androgens, cortisol, ACTH and thyroid function. Vitamin D deficiency and dyslipidemia were detected.

#### 3.5.2. Treatment

The patient was treated with exenatide LAR 2 mg/week for 6 months, with no changes in physical activity or diet, except for the last month, during which he started moderate physical activity, 1 h per week. The use of the elastic compression stocking (flat knit, second class) and the deep connective tissue massage sessions that she regularly performed every 6 weeks were not modified.

#### 3.5.3. Results

The patient was re-evaluated after 3 and 6 months of therapy. She had no side effects.

We observed a reduction of 6 kg in body weight in the first 3 months and a further reduction of 2 kg in the following 3 months (Table 6). The circumference of the abdomen and pelvis also progressively reduced.

The pain in the lower limbs, spontaneous and evoked by touch as well as the tendency to bruise, completely regressed. The total symptom score was reduced. The increase in one point observed for the total score at the end of the study is related to the increase in pain experienced after physical activity that the patient started in the last month.

The score of evoked pain in the lower limbs decreased in the first 3 months at all sites except for the lower medial third of the leg and the medial upper third of the thigh. After another 3 months, it decreased at all points except for the lower medial third of the leg and two sites on the thigh (medial lower third of the thigh and lateral upper third of the thigh).

Abdominal pain remained unchanged in the first 3 months, then decreased. In the upper limb, pain was reduced only in the forearm after 6 months of therapy (Table 6).

We also observed a normalization of tissue occurrence and a regression of pinching pain in the inframammary region, the anterior thorax. 

**Figure 5:**
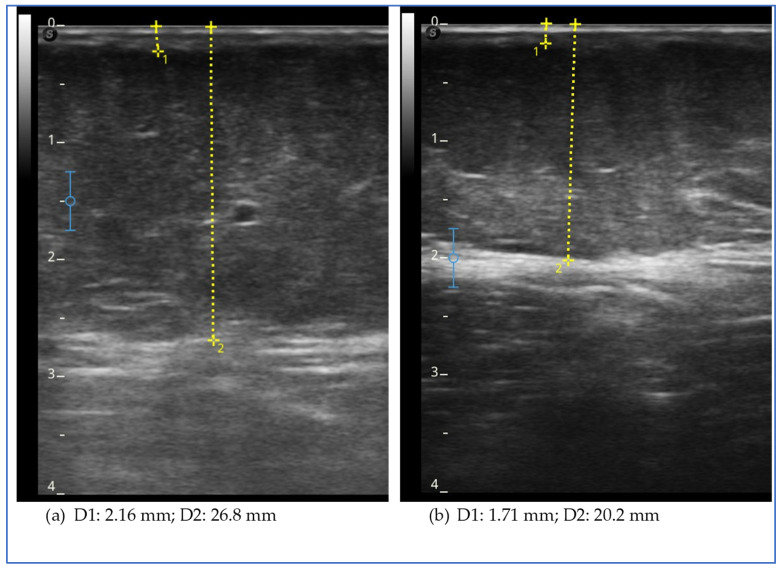
Ultrasound measurements for case report 5.

An ultrasound evaluation revealed a reduction in the thickness of the subcutaneous adipose tissue at all assessed levels of the lower limb and upper limb and abdomen, except for the medial upper third of the leg. The greatest reduction was observed in the medial thigh (Figure 5) and arm.

The figure shows the ultrasound measurements at the beginning of the study (a) and at the end of the study (b) for case report 5. The images refer to the medial upper third of the thigh. The yellow dotted line represents the measurement of the thickness of the skin, which includes the dermal and epidermal layers (D1), and of the subcutaneous adipose tissue (D2), which includes all the tissue from the skin to the muscular fascia.

## 4. Discussion

Insulin resistance is defined as a condition in which there is an inability of insulin-targeted tissue to respond to normal insulin levels, and thus, higher-than-normal levels of insulin are required to maintain the normal functions of insulin [3]. In recent years, the opposite hypothesis has also been postulated. It states that insulin resistance is a secondary phenomenon, an adaptive defense mechanism to a primary condition of hyperinsulinemia [4]. Both insulin resistance and hyperinsulinemia per se could be implicated in the case of lipedema, and both conditions could be either the consequence or the cause of a dysfunctional alteration in the adipose tissue [3,4,5,6]. The close physiological relationship between insulin and adipose tissue is an increasingly studied phenomenon because of its implications on human health, both in physiological and pathological conditions in which the biological cross talk is interfered by an unbalanced metabolic system or by dysfunctional adipose tissue. These phenomena, if persistent over time, tend to worsen and self-maintain. They involve adipose tissue as a whole and in all its functions, both as an endocrine organ and as an immune organ, determining local and systemic effects in a pro-inflammatory sense [3,4,5,6,7,8]. These phenomena are studied in the case of obesity but much less in the presence of lipedema, although, based on actual knowledge, they seem to be completely involved.

We presented five cases of patients with lipedema and insulin resistance who we treated with exenatide alone or in combination with modifications of diet and physical activity. We chose the cases of patients not treated with decongestive therapy because of possible greater interference in the results obtained.

We must acknowledge that it is not possible to know whether the observed outcomes can be attributed solely to pharmacological treatment because it is possible that the lifestyle changes implemented by the patients had a direct influence or favored all of the results described.

It is not possible with these few cases and without an adequate study protocol to exclude this aspect. To better comprehend this aspect, a more appropriate study (like a crossover study) could be conducted in the future.

At the time we started the pharmacological treatment of these patients, affected by both insulin resistance and obesity, no other GLP-1 RA or agonist of both GLP-1 and glucose-dependent insulinotropic polypeptide (GIP) with an indication for obesity treatment were available in Italy. The choice of exenatide was strongly supported by the data reported in the literature that showed, in addition to a good safety profile and good tolerability, positive effects on insulin resistance, body weight reduction, vascular and anti-inflammatory effects that were demonstrated both in patients with and without diabetes and with other chronic diseases [22,23,24,25,26,27,28,29,30,31,32,33,34,35]. We used once-weekly exenatide or exenatide long-acting release (LAR), available in this formulation in Italy for about 10 years. The long-acting formulation has proven to be well accepted by patients and easy to use with a maintained good safety profile while showing efficacy regarding metabolism and body weight control. Weekly dosing resulted in steady-state plasma exenatide concentrations after 6–7 weeks [48,49]. Our daily clinical experience, not limited to these five described cases, suggests a close relationship with the pharmacological profile: we are used to seeing a positive effect on exenatide therapy within the first 2–3 months of treatment. In our clinical practice, if no response is seen within these terms, continuation does not lead to important results, so we generally modify the therapeutic plan.

There are more interesting aspects that emerged from the results obtained that, in our opinion, should be considered, starting with the effect on body weight reduction. This effect, however, was not obtained in all cases: case report 1 did not achieve weight reduction. This patient was the one who had a lower degree of fasting insulin resistance (calculated with HOMA-IR) and the only one with a normal Matsuda index compared to the other cases.

This patient differs from the others in having less fasting insulin resistance and no insulin resistance, hyperinsulinemia, or glycemic abnormalities on OGTT. This suggests a possible connection between the severity of metabolic impairment and the response in terms of body weight reduction to the drug in the patient with lipedema. We cannot exclude the fact that glycemic levels influence the results; however, the most relevant metabolic alteration would appear to be insulin resistance and hyperinsulinemia. This hypothesis can also be supported by the marked weight reduction obtained in case 5, a case that differs from the others because it underwent previous surgery, especially because it is the case with greater hyperinsulinemia to OGTT.

Case report 1, however, is interesting precisely because it demonstrates an improvement in symptoms, clinical features and a reduction in adipose tissue thickness, even in the absence of weight loss. This result suggests a potential direct effect of exenatide on lipedematous tissue, also supported by the fact that the degree of insulin resistance in this patient was minimal. It is therefore possible to think that exenatide may have a role in the treatment of lipedema and that it could be mediated not only by the metabolic effect on insulin resistance, but also by other effects already reported in the literature involving the inflammatory and vascular states. As this is an inflammatory adipose tissue disorder, it is hypothesized that this effect is mediated by the reduction in systemic and tissue inflammation that GLP-1 drugs have been shown to cause in other inflammatory and chronic diseases, such as fatty liver disease and inflammatory bowel disease [30,31,32,33,34].

Focusing on the response to pain, we can see that spontaneous pain in the lower limbs has not improved, while provoked pain has reduced. This could be explained by the fact that spontaneous pain is more variable, which is why we prefer to evaluate all the symptoms together with the questionnaire. Even if it is easier and quicker to obtain a reduction in the spontaneous pain evaluation compared to the pain detected clinically with the tissue fold, this symptom can also be influenced by numerous other factors, including the emotional state, the phase of the menstrual cycle and even incorrect lifestyle habits [48,49,50].

Weight reduction was achieved in all other cases, both in association with or without an association with changes in the nutritional plan. The reduction in body weight in the other four patients reflects what has already been reported in the literature in patients with type 2 diabetes. In fact, the literature states that body weight reduction is progressive in the first 3 months and, subsequently, it continues in a more slowly way [51,52]. Even though there are only a few cases, the percentage reduction in weight obtained in the first 3 months of treatment, from 4.5% to 11.2%, in patients suffering from lipedema would appear to be potentially even greater than what is reported in the literature, which is approximately 2.5–3.4% in the first 3 months and 3.6% after 6 months in patients with type 2 diabetes mellitus [51,52]. The patient who showed the greatest percentage reduction is the one who was treated in combination with a ketogenic diet and then quickly regained weight with the reintroduction of carbohydrates (case 4). From this case, we could deduce at least two observations: The first is that exenatide could promote weight reduction following VLCD given that the patient had previously followed the same diet without obtaining any results, or simply that the effect of exenatide was enhanced by the current diet. The second observation, however, involves an alert relative to the excess weight produced and the excess speed in which it was reduced, with an immediate rebound effect upon the reintroduction of carbohydrates. Based on our experience, we observed that marked and sudden weight fluctuations with a yo-yo effect should be avoided both for the potential negative effects on adipose tissue and metabolic consequences, including hyperinsulinemia and elevated basal insulin secretion [53,54], but also because the goal of treating lipedema is to obtain positive results that can be maintained over time given the chronicity of the disease. However, it is also true that, in this case, the improvement in the subjective symptomatology and the clinical picture continued to improve despite the recovery of body weight. There is, however, one last observation for this case, which is more specific but must be highlighted: the exacerbation of the underlying psychiatric condition that the patient suffered during the combined treatment period and the marked reduction in weight. The reported effect was transitory and did not require changes in psychiatric therapy; however, it could be an aspect that must not be underestimated in the choice of a personalized therapeutic path.

A marked reduction in body weight was also observed in another patient who did not change her lifestyle and who, unlike the others, was the only one who had previously undergone liposuction. In this case, one might also think that the effect could have been facilitated by the fact that the increase in body weight and the worsening of lipedema were more recent compared to the other cases. It can therefore be assumed that the adipose tissue may have different characteristics, perhaps with less compromise of the adipose tissue, perhaps with less inflammatory, with less vascular compromise and a fibrotic component, but this is only a hypothesis. Although these observations were made in a single case, the extent of the clinical response, not only in terms of weight reduction, suggests the continued efficacy of the therapies and the potential use of these drugs in the post-operative phase. Also, from our daily experience, after surgery, there might be a possibility of relapses, or the disease may progress in untreated body areas. In fact, although targeted scientific studies are necessary, growth of tissue outside of the areas treated with surgery was reported in over 50% of patients of women with stage 2 or 3 lipedema and lipo-lymphedema. A total of 61% of patients noted new tissue growth within the first 6 months. In the same study, tissue growth in the areas treated with surgery was reported in approximately 30% of patients [55].

A reduction in waist circumference was obtained in all cases except for case 2, while in all cases, we observed a reduction, albeit variable, in pelvic circumference.

Focusing specifically on the symptoms reported by patients in the lower limbs, the results were consistent: in all cases, we observed a reduction in symptoms after the first 3 months of treatment independently of weight reduction or associated treatments.

The same result was obtained by evaluating the variation in pain evoked by the adipose tissue fold: in all cases, a reduction in the total score was obtained, with a reduction in both the score of the lower part of the body and of the upper part of the body. In our experience, this result is of particular clinical relevance, not always being consistent with the improvement in subjective symptoms.

In fact, in clinical practice, we have often observed that, despite a striking reduction in subjective symptoms, such as after a period of diet or halfway through decongestive treatment, clinically evoked lipoalgia remains markedly evocable [56]. It is not said that clinical findings, such as clinically evoked lipoalgia, are more important than subjective symptoms. However, in our opinion, the pain evoked by the tissue fold is fundamental in deciding whether to continue or modify the therapeutic plan. There are no available studies that have evaluated the effect of exenatide on these parameters, neither in patients with diabetes nor in patients without diabetes. This data is specific for lipedema, and with the same method, it has only been evaluated in our previous study following the use of a stocking with a micro massage effect not associated with changes in diet and in the absence of changes in body weight; even in this case, a reduction in the score of subjective symptoms in the lower limbs and the score of evoked lipoalgia in the lower limbs was obtained. However, no significant difference in evoked pain was observed in the untreated area of the upper body [36]. In the case of pharmacological treatment, the positive effect would appear to be systemic, i.e., more generalized than with local treatment, as expected.

The evaluation of the effect in this study is also supported by the observation of the modification of the thickness of the adipose tissue in specific points of the lower limbs, abdomen and upper limbs. This measurement, theoretically, can be more specific to study the effect on the subcutaneous tissue rather than what we can deduce on this tissue by observing the circumference of the limb or of the abdomen. However, it is not free from possible measurement errors and imprecision due to the ultrasound technique considering the difficulty in finding safe landmarks and the anatomical variation of each patient. In our opinion, it is of great help in clinical practice to evaluate the effects of all treatments, both conservative and surgical, and its use should be suggested and encouraged. In the five cases described, at the level of the lower limbs, we obtained a reduction in thickness in all patients and at all points evaluated, with the exception of three isolated points of different cases (the lateral lower third of the thigh in case 2, the lower lateral third of the leg in case 3 and the medial upper third of the leg in case 5). Case report 2 was the only one in which we did not observe a reduction in the thickness of the subcutaneous tissue at the level of the upper abdomen, while in all cases, a reduction in thickness was observed in the lower abdomen, an area often involved in lipedema. The presence of pain and the characteristics of the adipose tissue suggested that the lower abdomen was also involved in lipedema, with the exception of case 2. Therefore, the lack of tissue reduction in this case could be significant. However, further data are needed to verify the hypothesis. A reduction in upper limb thickness was variably observed in all cases described, except for case report 1. The reduction in upper limbs was particularly marked in the patient who had previously undergone liposuction of the lower limbs; it could be assumed that the tissue response to treatment may be more evident due to the recent clinical worsening. This data suggests a possible role of exenatide in patients with lipedema affected by insulin and who are undergoing surgery in the case of recurrence or clinical worsening.

## 5. Conclusions

We described the achievement of positive effects on the symptoms and signs of lipedema with a pharmacological treatment based on exenatide, a GLP-1 receptor agonist.

The results were demonstrated with both clinical and instrumental evaluations. Exenatide may promote weight loss, with an effect that appears to be related to the degree of insulin resistance. These effects become evident within the first months of treatment. The drug has been shown to reduce symptoms and signs of lipedema, even in the absence of a change in body weight, suggesting the presence of an additional extra-metabolic mechanism. These findings also show promising results for GLP1 Ras’ ability to manage recurrences or evolution of lipedema in patients who have previously undergone surgical interventions.

The results and observations presented in this study are certainly limited by several factors, including the sample size, the lack of homogeneity between patients and control groups and the possible imprecision in the results and measurements. However, they represent a promising starting point to also consider a pharmacological course of action in the treatment of the disease. They also underline the importance of an accurate diagnosis, firstly of lipedema and secondly of all comorbidities, to endorse multidisciplinary management of the patient, which has to include an endocrine–metabolic point of view.

## Figures and Tables

**Table 1:** Characteristics of the 5 clinical cases reported in the study. The table shows the clinical data and biochemical characteristics of the 5 case reports described in the study. The type of lipedema and the stage in the two districts, upper limbs and lower limbs, are reported. The table shows fasting blood glucose, insulin levels, calculated Homeostasis Model Assessment of Insulin Resistance (HOMA-IR) and values of glucose and insulin 60 min (60 min OGTT) and 120 min (120 min OGTT) after the 75 g Oral Glucose Tolerance Test (OGTT). The Area Under the Curve (AUC), calculated with the trapezoidal method, from 0 to 180 min (AUC 0–180) for glucose and for insulin is reported.

	Case Report 1	Case Report 2	Case Report 3	Case Report 4	Case Report 5
Age (yrs)	42	41	37	43	45
Body mass index (kg/m^2^)	31.5	36.5	38.1	37.8	30.1
Waist circumference (cm)	101	99	107	102	100
Hip circumference (cm)	126	122.5	128	112	111
Waist-to-hip ratio	0.80	0.81	0.84	0.91	0.91
Stage of lipedema—lower limbs	2	2	3	2	1
Type of lipedema—lower limb	3	3	3	3	3
Stage of lipedema—upper limbs	1	2	2	3	2
Type of lipedema—upper limbs	4A	4A	4A	4A	4C
Onset of lipedema (years)	13	10	6	30	18
Anterior pretibial fovea	Absent	Absent	Absent	Absent	Absent
Positive Stemmer’s sign	Absent	Absent	Absent	Absent	Absent
Telangiectasia	Absent	Present	Present	Present	Present
Acanthosis nigricans	Present	Present	Present	Present	Absent
Dorsal hump	Present	Present	Absent	Absent	Absent
Basal glucose OGTT (mg/dL)	100	110	94	109	89
Glucose 60 min OGTT (mg/dL)	120	149	114	192	181
Glucose 120 min OGTT (mg/dL)	122	131	93	176	134
Basal insulin OGTT (uIU/mL)	10.2	15	26.1	32.9	20.16
Insulin 60 min OGTT (uIU/mL)	43.0	70.4	171	136	226
Insulin 120 min OGTT (uIU/mL)	49.8	67.0	128	184	191
HOMA-IR	2.42	4.07	6.06	8.85	4.44
Matsuda index	4.97	2.95	1.61	1.14	1.50
Glucose AUC 0–180 min OGTT	20,280	24,195	18,765	29,865	25,275
Insulin AUC 0–180 min OGTT	6087	9564	24,913	25,639	28,619
HbA1c (mmol/mol)	31	35	31	-	36
Drug therapy	None	Levothyroxine	LNg IUD	fluoxetine, rosuvastatin, ezetimibe, mirtazapine	None

LNg IUD: Levonorgestrel-releasing intrauterine device. HbA1c: Glycosylated hemoglobin A1c.

**Table 2:** Case report 1. The table shows the anthropometric parameters, the lower limb symptom score measured with a questionnaire, the pain score evoked by the adipose tissue fold and the adipose tissue thickness measured with the ultrasound of case report 1. The data before (T0) and after 3 months of therapy (T3) and the changes (Delta T3–T0) are reported. The patient was treated with exenatide LAR 2 mg/week for 3 months in association with moderate physical activity (2 h/week). The patient did not change her diet. RPS: Ricolfi–Patton Score.

Parameters	T0	T3	Delta (T3–T0)
Body weight (kg)	89	89	0
Waist circumference (cm)	101	98	−3
Hip circumference (cm)	126	125	−1
Symptoms ^1^			
Spontaneous pain in the lower limbs	3	3	0
Feeling of swelling in the lower limbs	3	3	0
Pressure pain in the lower limbs	4	2	−2
Ease of bruising	3	1	−2
Total symptom score	52	36	−16
Evoked pain or lipoalgia ^2^			
Lower medial third of the leg	4	3	−1
Posterior middle third of the leg	3	3	0
Medial upper third of the leg	4	2	−2
Medial lower third of the thigh	4	4	0
Medial upper third of the thigh	4	4	0
Lateral upper third of the thigh	4	3	−1
Lateral lower third of the thigh	4	3	−1
Lower abdomen	3	1	−2
Lateral subscapular region	3	1	−2
Arm	2	3	+1
Forearm	0	0	0
Lower body pain score	30	23	−7
Upper body pain score	5	4	−1
Total body pain score or RPS	36	27	−9
Thickness of subcutaneous tissue ^3^			
Lower medial third of the leg (mm)	18.4	13.3	−5.1
Medial upper third of the leg (mm)	9.6	7.21	−2.39
Medial lower third of the thigh (mm)	33.3	27.6	−5.7
Medial upper third of the thigh (mm)	36.3	28.6	−7.7
Upper anterior third of the thigh (mm)	13.8	11.2	−2.6
Anterior middle third of the thigh (mm)	24.5	22.0	−2.5
Lateral lower third of the thigh (mm)	29.1	26.5	−2.6
Upper lateral third of the leg (mm)	11.4	8.03	−3.37
Lower lateral third of the leg (mm)	11.4	8.70	−2.7
Lower abdomen (mm)	35.3	27.3	−8
Upper abdomen (mm)	35.3	29.0	−6.3
Midpoint upper arm (mm)	12.4	15.7	+3.3
Midpoint forearm (mm)	6.21	5.8	−0.41

^1^ Symptoms are reported on a 6-point Likert scale (0 = none, 1 = very mild, 2 = mild, 3 = moderate, 4 = severe and 5 = very severe). The total symptom score is the sum of the individual scores for the 17 symptom items of the questionnaire (ranging from 0 to 85). ^2^ Pain evoked, or lipoalgia, was scored on a numeric rating scale from 0 to 4 (0 = no pain, 1 = mild pain, 2 = moderate pain, 3 = severe pain and 4 = very severe pain). The lower body pain score is the sum of the lower limb and abdomen scores (ranging from 0 to 32). The upper body pain score is the sum of the scores for the upper limbs and back (ranging from 0 to 12). The total body pain score or Ricolfi–Patton Score (RPS) is the sum of the value of all points (ranging from 0 to 44). ^3^ The thickness of the subcutaneous tissue was measured by ultrasound and includes all the tissue, from the skin to the muscular fascia.

**Table 3:** Case report 2. The table shows the anthropometric parameters, the lower limb symptom score measured with a questionnaire, the pain score evoked by the adipose tissue fold and the adipose tissue thickness measured with the ultrasound of case report 2. The data before (T0) and after 3 months of therapy (T3) and the changes (Delta T3–T0) are reported. The patient was treated with exenatide LAR 2 mg/week for 3 months in combination with moderate physical activity (3 h/week) and a reduction in the daily consumption of sweets and refined sugar. RPS: Ricolfi–Patton Score.

Parameters	T0	T3	Delta (T3–T0)
Body weight (kg)	87	82	−5
Waist circumference (cm)	99	99	0
Hip circumference (cm)	123	120	−3
Symptoms ^1^			
Spontaneous pain in the lower limbs	3	1	−2
Feeling of swelling in the lower limbs	2	1	−1
Pressure pain in the lower limbs	4	1	−3
Ease of bruising	1	1	0
Total symptom score	36	17	−9
Evoked pain or lipoalgia ^2^			
Lower medial third of the leg	2	2	0
Posterior middle third of the leg	2	2	0
Medial upper third of the leg	4	3	−1
Medial lower third of the thigh	4	3	−1
Medial upper third of the thigh	4	3	−1
Lateral upper third of the thigh	2	1	−1
Lateral lower third of the thigh	4	1	−3
Lower abdomen	0	0	0
Lateral subscapular region	3	1	−2
Arm	3	1	−2
Forearm	0	0	0
Lower body pain score	22	15	−7
Upper body pain score	6	2	−4
Total body pain score or RPS	28	17	−11
Thickness of subcutaneous tissue ^3^			
Lower medial third of the leg (mm)	17.5	16.2	−1.3
Medial upper third of the leg (mm)	16.5	16.1	−0.4
Medial lower third of the thigh (mm)	38.1	33.0	−5.1
Medial upper third of the thigh (mm)	45.8	38.4	−7.4
Upper anterior third of the thigh (mm)	16.4	14.1	−2.3
Anterior middle third of the thigh (mm)	22.7	21.8	−0.9
Lateral lower third of the thigh (mm)	23.8	24.9	1.1
Upper lateral third of the leg (mm)	19.0	17.3	−1.7
Lower lateral third of the leg (mm)	5.41	5.16	−0.25
Lower abdomen (mm)	48.5	46.0	−2.5
Upper abdomen (mm)	35.6	39.9	4.3
Midpoint upper arm (mm)	20.1	19.6	−0.5
Midpoint forearm (mm)	8.04	5.90	−2.14

^1^ Symptoms are reported on a 6-point Likert scale (0 = none, 1 = very mild, 2 = mild, 3 = moderate, 4 = severe and 5 = very severe). The total symptom score is the sum of the individual scores for the 17 symptom items of the questionnaire (ranging from 0 to 85). ^2^ Pain evoked, or lipoalgia, was scored on a numeric rating scale from 0 to 4 (0 = no pain, 1 = mild pain, 2 = moderate pain, 3 = severe pain and 4 = very severe pain). The lower body pain score is the sum of the lower limb and abdomen scores (ranging from 0 to 32). The upper body pain score is the sum of the scores for the upper limbs and back (ranging from 0 to 12). The total body pain score or Ricolfi–Patton Score (RPS) is the sum of the value of all points (ranging from 0 to 44). ^3^ The thickness of the subcutaneous tissue was measured by ultrasound and includes all the tissue from the skin to the muscular fascia.

**Table 4:** Case report 3. The table shows the anthropometric parameters, the lower limb symptom score measured with a questionnaire, the pain score evoked by the adipose tissue fold and the adipose tissue thickness measured with the ultrasound of case report 3. The data before (T0), after 3 months (T3) and after 6 months of therapy (T6) and the variation (Delta T6–T0) are reported. The patient was treated with exenatide LAR 2 mg/week for 6 months in combination with moderate physical activity (3 h/week) and an anti-inflammatory, gluten-free, lactose-free, low-carb and hypocaloric diet. RPS: Ricolfi–Patton Score.

Parameters	T0	T3	T6	Delta (T6–T0)
Body weight (kg)	95	90.7	89	−6
Waist circumference (cm)	107	-	101	−6
Hip circumference (cm)	128	-	123	−5
Symptoms ^1^				
Spontaneous pain in the lower limbs	4	-	1	−3
Feeling of swelling in the lower limbs	5	-	3	−2
Pressure pain in the lower limbs	4	-	3	−1
Ease of bruising	3	-	1	−2
Total symptom score	50	-	32	−18
Evoked pain or lipoalgia ^2^				
Lower medial third of the leg	4	-	4	0
Posterior middle third of the leg	4	-	2	−2
Medial upper third of the leg	4	-	2	−2
Medial lower third of the thigh	4	-	3	−1
Medial upper third of the thigh	4	-	2	−2
Lateral upper third of the thigh	4	-	2	−2
Lateral lower third of the thigh	4	-	3	−1
Lower abdomen	1	-	0	−1
lateral subscapular region	4	-	2	−2
Arm	4	-	2	−2
Forearm	0	-	0	0
Lower body pain score	29	-	18	−11
Upper body pain score	8	-	4	−4
Total body pain score or RPS	37	-	22	−15
Thickness of subcutaneous tissue ^3^				
Lower medial third of the leg (mm)	26.7	-	25	−1.7
Medial upper third of the leg (mm)	16	-	15.7	−0.3
Medial lower third of the thigh (mm)	33.7	-	29.2	−4.5
Medial upper third of the thigh (mm)	34.3	-	25.1	−9.2
Upper anterior third of the thigh (mm)	15.4	-	12.4	−3
Anterior middle third of the thigh (mm)	-	-	-	-
Lateral lower third of the thigh (mm)	19.2	-	14.5	−4.7
Upper lateral third of the leg (mm)	17.2	-	13	−4.2
Lower lateral third of the leg (mm)	10.8	-	10.8	0
Lower abdomen (mm)	50.3	-	44.4	−5.9
Upper abdomen (mm)	49.6	-	38.9	−10.7
Midpoint upper arm (mm)	18.4	-	14.6	−3.8
Midpoint forearm (mm)	10.5	-	9.2	−1.3

^1^ Symptoms are reported on a 6-point Likert scale (0 = none, 1 = very mild, 2 = mild, 3 = moderate, 4 = severe and 5 = very severe). The total symptom score is the sum of the individual scores for the 17 symptom items of the questionnaire (ranging from 0 to 85). ^2^ Pain evoked, or lipoalgia, was scored on a numeric rating scale from 0 to 4 (0 = no pain, 1 = mild pain, 2 = moderate pain, 3 = severe pain and 4 = very severe pain). The lower body pain score is the sum of the lower limb and abdomen scores (ranging from 0 to 32). The upper body pain score is the sum of the scores for the upper limbs and back (ranging from 0 to 12). The total body pain score or Ricolfi–Patton Score (RPS) is the sum of the value of all points (ranging from 0 to 44). ^3^ The thickness of the subcutaneous tissue was measured by ultrasound and includes all the tissue from the skin to the muscular fascia.

**Table 5:** Case report 4. The table shows the anthropometric parameters, the lower limb symptom score measured with a questionnaire, the pain score evoked by the adipose tissue fold and the adipose tissue thickness measured with the ultrasound of case report 4. The data before (T0), after 3 months (T3) and after 6 months (T6) of therapy and the variation from the baseline (Delta T3–T0 and Delta T6–T0) are reported. The patient was treated with exenatide LAR 2 mg/week for 6 months in combination with VLCKD in the first 3 months, followed by a 3-month phase of gradual reintroduction of carbohydrates and a hypocaloric diet (gluten free). She did not change her physical activity. RPS: Ricolfi–Patton Score.

Parameters	T0	T3	T6	Delta (3–0)	Delta (6–0)
Body weight (kg)	87.3	77.5	84.8	−9.8	−2.5
Waist circumference (cm)	102	95	101	−7	−1
Hip circumference (cm)	112	107	114	−5	+2
Symptoms ^1^					
Spontaneous pain in the lower limbs	4	3	0	−1	−4
Feeling of swelling in the lower limbs	5	3	1	−2	−4
Pressure pain in the lower limbs	4	3	0	−1	−4
Ease of bruising	5	2	0	−3	−5
Total symptom score	69	38	8	−31	−61
Evoked pain or lipoalgia ^2^					
Lower medial third of the leg	4	2	0	−2	−4
Posterior middle third of the leg	4	0	0	−4	−4
Medial upper third of the leg	4	0	0	−4	−4
Medial lower third of the thigh	4	2	1	−2	−3
Medial upper third of the thigh	4	4	0	0	−4
Lateral upper third of the thigh	4	2	0	−2	−4
Lateral lower third of the thigh	4	0	0	−4	−4
Lower abdomen	2	0	0	−2	−2
lateral subscapular region	0	0	0	0	0
Arm	4	2	0	−2	−4
Forearm	0	0	0	0	0
Lower body pain score	30	10	1	−20	−29
Upper body pain score	4	2	0	−2	−4
Total body pain score or RPS	34	12	1	−22	−33
Thickness of the subcutaneous tissue ^3^					
Lower medial third of the leg (mm)	15.7	14.1	15.4	−1.6	−0.3
Medial upper third of the leg (mm)	19.8	16.3	16.3	−3.5	−3.5
Medial lower third of the thigh (mm)	47.8	44.3	49.0	−3.5	1.2
Medial upper third of the thigh (mm)	46.4	33.2	42.6	−13.2	−3.8
Upper anterior third of the thigh (mm)	16.2	16	15.7	−0.2	−0.5
Anterior middle third of the thigh (mm)	20.9	20.6	20.6	−0.2	−0.2
Lateral lower third of the thigh (mm)	19.4	15.8	17.5	−3.6	−1.9
Upper lateral third of the leg (mm)	7.54	6.05	6.74	−1.49	−0.80
Lower lateral third of the leg (mm)	5.12	3.27	4.65	−1.85	−0.47
Lower abdomen (mm)	53.5	45.5	46.2	−8.0	−7.3
Upper abdomen (mm)	45.9	35.8	34.2	−10.1	−11.7
Midpoint upper arm (mm)	22.9	20.7	22.1	−2.2	−0.8
Midpoint forearm (mm)	13.3	12.4	13.3	−0.6	0

^1^ Symptoms are reported on a 6-point Likert scale (0 = none, 1 = very mild, 2 = mild, 3 = moderate, 4 = severe and 5 = very severe). The total symptom score is the sum of the individual scores for the 17 symptom items of the questionnaire (ranging from 0 to 85). ^2^ Pain evoked, or lipoalgia, was scored on a numeric rating scale from 0 to 4 (0 = no pain, 1 = mild pain, 2 = moderate pain, 3 = severe pain and 4 = very severe pain). The lower body pain score is the sum of the lower limb and abdomen scores (ranging from 0 to 32). The upper body pain score is the sum of the scores for the upper limbs and back (ranging from 0 to 12). The total body pain score or Ricolfi–Patton Score (RPS) is the sum of the value of all points (ranging from 0 to 44). ^3^ The thickness of the subcutaneous tissue was measured by ultrasound and includes all the tissue from the skin to the muscular fascia.

**Table 6:** Case report 5. The table shows the anthropometric parameters, the lower limb symptom score measured with a questionnaire, the pain score evoked by the adipose tissue fold and the adipose tissue thickness measured with the ultrasound of case report 5. The data before (T0), after 3 months (T3) and after 6 months (T6) of therapy and the variation from the baseline (Delta T3–T0 and Delta T6–T0) are reported. The patient was treated with exenatide LAR 2 mg/week for 6 months, without changes in diet or compression stocking use. Last month, he started moderate physical activity, 1 h per week. RPS: Ricolfi–Patton Score.

Parameters	T0	T3	T6	Delta 3–0	Delta 6–0
Body weight (kg)	81	75	73	−6	−8
Waist circumference (cm)	107	90	82	−17	−25
Hip circumference (cm)	128	114	111	−14	−17
Symptoms ^1^					
Spontaneous pain in the lower limbs	1	0	0	−1	−1
Feeling of swelling in the lower limbs	1	0	0	−1	−1
Pressure pain in the lower limbs	2	0	0	−2	−2
Ease of bruising	0	0	0	0	0
Total symptom score	14	4	5	−10	−9
Evoked pain or lipoalgia ^2^					
Lower medial third of the leg	2	2	2	0	0
Posterior middle third of the leg	4	2	0	−2	−4
Medial upper third of the leg	4	3	2	−1	−2
Medial lower third of the thigh	4	3	4	−1	0
Medial upper third of the thigh	4	4	3	0	−1
Lateral upper third of the thigh	2	3	2	1	0
Lateral lower third of the thigh	3	2	2	−1	−1
Lower abdomen	2	2	1	0	−1
Lateral subscapular region	3	4	4	1	1
Arm	4	4	4	0	0
Forearm	1	0	0	−1	−1
Lower body pain score	25	21	16	−4	−9
Upper body pain score	8	8	8	0	0
Total body pain score or RPS	33	29	24	−4	−9
Thickness of the subcutaneous tissue ^3^					
Lower medial third of the leg (mm)	15.2	15.20	13.20	0	−2
Medial upper third of the leg (mm)	8.89	12.20	9.22	3.31	0.33
Medial lower third of the thigh (mm)	31.8	18.80	16.70	−13	−15.1
Medial upper third of the thigh (mm)	26.8	20.60	20.20	−6.2	−6.6
Upper anterior third of the thigh (mm)	10.6	7.83	9.01	−2.77	−1.59
Anterior middle third of the thigh (mm)	-	14.00	11.90	-	-
Lateral lower third of the thigh (mm)	19.3	14.90	12.90	−4.4	−6.4
Upper lateral third of the leg (mm)	9.5	9.64	9.39	0.14	−0.11
Lower lateral third of the leg (mm)	17.5	16.00	15.90	−1.5	−1.6
Lower abdomen (mm)	32.8	32.20	27.60	−0.6	−5.2
Upper abdomen (mm)	22	21.10	18.20	−0.9	−3.8
Midpoint upper arm (mm)	25.6	18.00	14.40	−7.6	−11.2
Midpoint forearm (mm)	13.9	11.6	10.80	−2.3	−3.1

^1^ Symptoms are reported on a 6-point Likert scale (0 = none, 1 = very mild, 2 = mild, 3 = moderate, 4 = severe and 5 = very severe). The total symptom score is the sum of the individual scores for the 17 symptom items of the questionnaire (ranging from 0 to 85). ^2^ Pain evoked, or lipoalgia, was scored on a numeric rating scale from 0 to 4 (0 = no pain, 1 = mild pain, 2 = moderate pain, 3 = severe pain and 4 = very severe pain). The lower body pain score is the sum of the lower limb and abdomen scores (ranging from 0 to 32). The upper body pain score is the sum of the scores for the upper limbs and back (ranging from 0 to 12). The total body pain score or Ricolfi–Patton Score (RPS) is the sum of the value of all points (ranging from 0 to 44). ^3^ The thickness of the subcutaneous tissue was measured by ultrasound and includes all the tissue from the skin to the muscular fascia.

## Data Availability

The derived data supporting the findings of this study are available from the corresponding authors upon reasonable request.

## Abbreviations

The following abbreviations are used in this manuscript:

GLP-1 RAs	Glucagon-Like Peptide-1 Receptor Agonists
exenatide LAR	Exenatide Long-Acting Release
OGTT	Oral Glucose Tolerance Test
AUC	Area Under the Curve
HbA1c	Glycosylated Hemoglobin A1c
VLCKD	Very-Low-Calorie Ketogenic Diet
MCP-1	Monocyte Chemoattractant Protein-1
TNF-alpha	Tumor Necrosis Factor alpha
IL-1 beta	Interleukin-1beta
HOMA-IR	Homeostasis Model Assessment of Insulin Resistance
LNg IUD	Levonorgestrel-Releasing Intrauterine Device
RPS	Ricolfi–Patton Score
VRS	Verbal Rating Scale
PPC	Progressive Pain Check
LBPS	Lower Body Pain Score
UBPS	Upper Body Pain Score
